# Cardiac Efficiency and Work Performance Variations Across Menstrual Cycle Phases: A Bicycle Ergometric Study in Young Women

**DOI:** 10.7759/cureus.78216

**Published:** 2025-01-29

**Authors:** R Lavanya, R A Sureshbalaji, S Prem Kumar

**Affiliations:** 1 Physiology, Saveetha Medical College and Hospital, Chennai, IND; 2 Physiology, Vethathiri Maharishi College of Yoga, Chennai, IND; 3 Physiology, Trichy SRM Medical College Hospital and Research Centre, Tiruchirappalli, IND; 4 Physiology, Madha Medical College and Research Institute, Chennai, IND

**Keywords:** bicycle ergometric test, cardiac efficiency, follicular phase, luteal phase, menstrual cycle, work capacity evaluation

## Abstract

Background

Menstrual cycle-related physiological variations represent a complex, multifaceted phenomenon with significant implications for female work performance and cardiovascular function. This study aimed to systematically evaluate the influence of menstrual cycle phases on cardiac efficiency and work performance among young women, utilizing a comprehensive bicycle ergometric assessment methodology. The research sought to quantify physiological variations during mid-follicular and mid-luteal phases, providing nuanced insights into hormonal dynamics and performance metrics.

Methodology

A prospective observational study was conducted among 100 young women volunteers aged 18-25 years in Chennai, Tamil Nadu, India. Participants underwent standardized bicycle ergometer testing during two distinct menstrual cycle phases: mid-follicular (seventh day) and mid-luteal (21st day). A bicycle ergometer (KH-695, Viva Fitness Company, New Delhi, India) was employed to measure energy expenditure, work performance, and cardiac efficiency. Subjects initially underwent a five-minute resting period, with baseline pulse rate and blood pressure recorded. Participants then performed cycling at a 2 kg resistance for a maximum of five minutes, with pulse rates monitored during post-exercise recovery intervals. Cardiac efficiency was calculated using a comprehensive formula incorporating exercise duration and post-exercise pulse rates, while work done was determined through precise mechanical measurements.

Results

Statistical analysis revealed significant physiological variations across menstrual cycle phases. Cardiac efficiency demonstrated a remarkable increase from 79.98 (SD ± 17.618) in the mid-follicular phase to 112.58 (SD ± 13.086) in the mid-luteal phase, with 95 out of 100 participants exhibiting enhanced performance (Z-statistic = -8.625, p = 0.000). Total work done similarly showed substantial improvements, increasing from 185.77 (SD ± 35.82) to 242.97 (SD ± 31.275), with 97 observations indicating superior luteal phase performance (Z-statistic = -8.374, p = 0.000). Notably, work done per minute remained consistently stable across both phases, suggesting an intrinsic physiological adaptation mechanism. The Wilcoxon signed-rank test confirmed statistically significant differences in cardiac efficiency and total work done, highlighting the complex interplay between hormonal fluctuations and physiological performance.

Conclusions

The study demonstrates significant menstrual cycle phase-related variations in cardiac efficiency and work performance, providing crucial insights into female physiological adaptability and underscoring the importance of personalized performance management strategies across different reproductive cycle stages.

## Introduction

The complex physiological variations associated with the menstrual cycle have long intrigued researchers in the domains of exercise physiology, reproductive biology, and occupational health. The cyclical hormonal fluctuations experienced by women can potentially modulate various physiological parameters, including cardiovascular function, metabolic performance, and exercise capacity [1,2]. Understanding these nuanced interactions becomes crucial in comprehending how menstrual cycle phases might influence work performance and physiological efficiency.

Existing literature suggests that hormonal variations during different menstrual cycle phases can significantly impact physiological responses and exercise performance [3]. Hormones such as estrogen and progesterone demonstrate substantial effects on cardiovascular regulation, metabolic processes, and neuromuscular functioning [4-6]. These hormonal dynamics potentially create differential responses in cardiac efficiency, work capacity, and overall physiological resilience across distinct menstrual cycle phases [7,8].

Previous research has explored the multifaceted implications of menstrual cycle phases on various performance metrics. Systematic reviews and meta-analyses have consistently highlighted the potential variations in physical performance during different menstrual cycle stages [1,2,9]. Investigations have demonstrated that athletic performance, muscular strength, endurance, and metabolic responses can exhibit notable fluctuations corresponding to hormonal variations [10-12].

The workplace productivity implications of menstrual cycle-related physiological changes have also garnered increasing research attention. Recent cross-sectional surveys have indicated substantial productivity losses associated with menstruation-related symptoms, underscoring the critical need for a comprehensive understanding of these physiological dynamics [13,14]. Furthermore, psychological responses and exercise adherence have been explored, revealing potential interconnections between menstrual cycle phases and performance variability [15,16].

Cardiac efficiency and work performance represent complex, multidimensional constructs influenced by numerous physiological parameters. While prior studies have investigated isolated aspects of menstrual cycle impacts, comprehensive assessments utilizing objective measurement techniques remain limited [17-19]. The present study aims to bridge this research gap by employing a rigorous bicycle ergometric approach to systematically evaluate cardiac efficiency and work performance across mid-follicular and mid-luteal menstrual cycle phases.

By utilizing precise measurement techniques and a standardized experimental protocol, this research seeks to provide nuanced insights into the physiological variations experienced by young women during different menstrual cycle stages. The findings can potentially inform exercise prescription, occupational health strategies, and individual performance optimization approaches.

## Materials and methods

Study design and setting

This prospective observational study was conducted among women volunteers at Saveetha Medical College and Hospital located in Chennai, Tamil Nadu, a state in southern India. By selecting a tertiary care center in Chennai as the research site, the study leveraged sophisticated medical infrastructure. Tamil Nadu, known for its advanced healthcare system, provided an optimal environment for conducting rigorous scientific research with high-quality medical facilities and professional expertise.

Study duration

The research was implemented over a comprehensive 12-month period, spanning from January 2021 to December 2021.

Sample size estimation and sampling method

A cross-sectional study by Ponzo et al. in the United States revealed that 68.3% of participants reported moderate to severe negative impacts on work performance [13]. We have calculated the sample size using the following formula: $N = \frac{3.84 \times p \times q}{d^2}$,

where p represents the prevalence (68.3%), q represents the complement of prevalence (31.7%), and d represents the precision (10% absolute error). Through this calculation, the study required a minimum of 84 participants to ensure statistical reliability and representativeness. To enhance statistical robustness and account for potential data variability, we expanded the sample to 100 women. Convenient sampling was utilized to recruit 100 participants, allowing for efficient subject selection while maintaining the study's methodological integrity. The age range of 18-25 years was strategically chosen to minimize potential confounding factors related to physiological variations across different life stages.

Inclusion criteria

Women of 18-25 years with a regular 28-day menstrual cycle and normal blood pressure were considered eligible for participation.

Exclusion criteria

Subjects with any cardiorespiratory diseases were systematically excluded to prevent potential interference with cardiac efficiency measurements. This criterion ensures that the study’s physiological assessments reflect baseline health parameters. Participants with epilepsy or active infectious diseases were precluded from the study. These conditions could significantly alter physiological responses and compromise the accuracy of performance metrics. Specific gynecological conditions were also grounds for exclusion. They were irregular menstruation, dysmenorrhea, and polycystic ovarian disease.

Data collection procedure

A semi-structured interview schedule administered by an interviewer was used to collect data on age, menstrual, and medical history. Physiological measurements were conducted using specialized equipment, including a bicycle ergometer (KH-695, Viva Fitness Company, New Delhi, India) for work performance assessment, a sphygmomanometer for blood pressure measurement, and a stopwatch for manual pulse rate tracking. Anthropometric measurements were also collected to calculate BMI.

The bicycle ergometer served as a stationary exercise apparatus specifically designed for detailed physiological research. This instrument provides comprehensive measurement capabilities, including energy expenditure tracking in calories, precise pulse rate monitoring, and exercise duration assessment. The ergometer’s design incorporates adjustable resistance settings and flexible bicycle height configuration, enabling personalized accommodation of individual participant characteristics.

The observational protocol was meticulously structured around two critical menstrual cycle phases: the mid-follicular phase (seventh day) and the mid-luteal phase (21st day). Each assessment followed a standardized procedure beginning with a five-minute resting period, during which baseline pulse rate and blood pressure were recorded. Participants then engaged in a cycling exercise at a consistent 2 kg resistance, representing moderate-intensity physical exertion. The exercise was limited to a maximum duration of five minutes or until participants reported fatigue.

Following the exercise intervention, researchers conducted systematic post-exercise measurements. Pulse rates were carefully recorded at one-minute intervals during the first, second, and third minutes after exercise. Coinciding with pulse rate measurements, blood pressure was also documented to provide a comprehensive physiological profile. This methodical approach allowed for precise calculation of cardiac efficiency, work performance, and other critical physiological parameters.

Calculation methodologies were rigorously defined to ensure data reliability. Cardiac efficiency was computed using a formula that considered exercise duration and post-exercise pulse rates:

$Cardiac\ Efficiency = \frac{Duration_{exercise}\ (seconds) \times 100}{\sum_{i=1}^{3} PR_{i}}$,

where PR represents post-exercise pulse rate measurements at minutes 1, 2, and 3.

Work done measurements incorporated calculations involving wheel circumference, tension, and rotational parameters:

Work done = wheel circumference × tension × rotation per minute,

where wheel circumference = 2 π r; radius of the wheel (r) = 12.5 cm.

The BMI is calculated using the following formula: $BMI = \frac{weight_{kg}}{height_{m}^2}$

Ethics committee approval

The research protocol received formal approval from the Institutional Ethics Committee of a tertiary care center located in Chennai (approval number SMC/IEC/2020/03/046). This approval demonstrates adherence to established ethical standards in medical research. Prior to the study’s commencement, each participant underwent a comprehensive informed consent procedure. Researchers meticulously explained the study’s objectives and methodological procedures in the participant’s preferred language, ensuring complete understanding and voluntary participation. Participant confidentiality was maintained throughout the study, and data were stored securely following standard research protocols.

Data analysis

The data gathered was entered into Microsoft Excel (Microsoft Corporation, Redmond, WA, USA). Statistical analyses were performed using IBM SPSS Statistics for Windows, Version 23.0 (Released 2015; IBM Corp., Armonk, NY, USA). Continuous variables in this study were comprehensively characterized using multiple statistical descriptors: mean, median, SD, and IQR. The Shapiro-Wilk test was employed to evaluate the normal distribution of continuous variables. This statistical test helps researchers determine whether parametric or nonparametric analytical techniques are most appropriate for subsequent data analysis. To compare physiological parameters between luteal and follicular phases, we utilized the Wilcoxon signed-rank test, a nonparametric method ideal for paired comparisons when data do not conform to normal distribution. This test allows for robust statistical inference without assuming normality. A two-tailed p-value of <0.05 was considered statistically significant for all analyses.

## Results

The study population consisted of 100 young women with a mean age of 22.71 years (SD ± 1.192), characterized by a median age of 23 years and an IQR of 22-24 years. The participants demonstrated relatively consistent anthropometric characteristics. The mean body weight was 59.98 kg (SD ± 5.73), with a median of 60 kg and an IQR of 55-64 kg. The mean height was 1.621 meters (SD ± 0.066), with a median of 1.62 meters and an IQR of 1.57-1.67 meters. The mean BMI was 22.86 kg/m² (SD ± 2.121), with a median of 22.713 and an IQR of 21.46-24.08 kg/m². Table 1 shows the demographic and anthropometric characteristics of study participants.

**Table 1:** Demographic and anthropometric characteristics of study participants ^*^ A Shapiro-Wilk test was used to detect the normality and was considered statistically significant if the p-value was less than 0.05. Normality testing using the Shapiro-Wilk test revealed statistically significant deviations from normal distribution for age (p = 0.000), height (p = 0.045), and BMI (p = 0.018), suggesting potential nonparametric characteristics for these variables. In contrast, weight demonstrated a non-significant deviation from normality (p = 0.102), indicating a more normally distributed characteristic.

Characteristics	Mean ± SD	Median (IQR)	p-value
Age	22.71 ± 1.192	23 (22-24)	0.000^*^
Weight in kg	59.98 ± 5.73	60 (55-64)	0.102
Height in m	1.6209 ± 0.066	1.62 (1.57-1.67)	0.045^*^
BMI (kg/m²)	22.859 ± 2.1208	22.713 (21.46-24.08)	0.018^*^

The physiological performance parameters during the mid-follicular phase revealed notable characteristics across three key metrics, as shown in Table 2. Cardiac efficiency demonstrated a mean value of 79.98 (SD ± 17.618), with a median of 78 and an IQR of 68-87.75. Total work done exhibited a mean of 185.77 (SD ± 35.82), with a median of 179.5 and an IQR of 169-196. Work done per minute showed a relatively consistent performance, with a mean of 59.26 (SD ± 2.46), a median of 60, and an IQR of 58-61.

**Table 2:** Physiological performance metrics during mid-follicular phase: descriptive and normality analysis ^* ^A Shapiro-Wilk test was used to detect the normality and was considered statistically significant if the p-value was less than 0.05. The Shapiro-Wilk normality test indicated statistically significant deviations from a normal distribution for all three metrics (p = 0.000), suggesting potential nonparametric characteristics for these physiological variables.

Follicular phase	Mean ± SD	Median (IQR)	p-value
Cardiac efficiency	79.98 ± 17.618	78 (68-87.75)	0.000^*^
Total work done	185.77 ± 35.82	179.5 (169-196)	0.000^*^
Work done per min	59.26 ± 2.46	60 (58-61)	0.000^*^

The physiological performance parameters during the mid-luteal phase revealed distinct characteristics across three key metrics, as shown in Table 3. Cardiac efficiency demonstrated a mean value of 112.58 (SD ± 13.086), with a median of 110.5 and an IQR of 104-124.75. This represents a notable increase in cardiac efficiency compared to the mid-follicular phase. Total work done showed a mean of 242.97 (SD ± 31.275), with a median of 241 and an IQR of 222.25-264.75. Work done per minute remained remarkably consistent, with a mean of 59.37 (SD ± 2.501), a median of 60, and an IQR of 58-61.

**Table 3:** Physiological performance metrics during mid-luteal phase: descriptive and normality analysis ^*^ The total work done metric exhibited a more normally distributed pattern, as evidenced by the Shapiro-Wilk test p-value of 0.221, which suggests no significant deviation from normal distribution. However, the Shapiro-Wilk normality test indicated a statistically significant deviation from a normal distribution (p < 0.05) for cardiac efficiency and work done per minute.

Luteal phase	Mean ± SD	Median (IQR)	p-value
Cardiac efficiency	112.58 ± 13.086	110.5 (104-124.75)	0.001^*^
Total work done	242.97 ± 31.275	241 (222.25-264.75)	0.221
Work done per min	59.37 ± 2.501	60 (58-61)	0.000^*^

**Figure 1:**
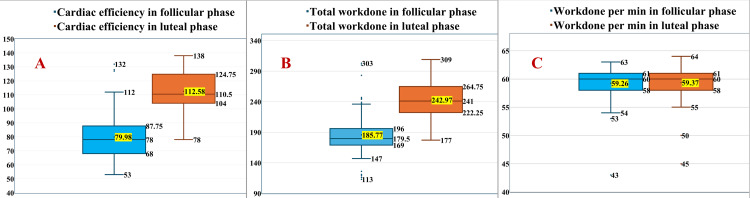
Comparative analysis of cardiac parameters during follicular and luteal phases of the menstrual cycle Panel A illustrates cardiac efficiency variations, panel B depicts total work done (113-309 units), and panel C demonstrates work done per minute. Blue box plots: follicular phase measurements; Orange box plots: luteal phase measurements

Figure 1 presents the box and whisker plot of cardiac parameters across menstrual cycle phases in three panels. Panel A illustrates cardiac efficiency variations (53-138 units), panel B depicts total work done (113-309 units), and panel C demonstrates work done per minute (45-64 units). The data consistently indicates elevated values during the luteal phase compared to the follicular phase, suggesting significant physiological adaptations between phases.

The Wilcoxon signed-rank test revealed significant differences between the luteal and follicular phases across multiple physiological parameters, as shown in Table 4. Cardiac efficiency showed a marked disparity, with 95 out of 100 observations indicating higher performance during the luteal phase, compared to only five observations showing lower efficiency. The Z statistic of -8.625 and an extremely low p-value of 0.000 suggest a statistically significant difference in cardiac efficiency between these menstrual cycle phases.

**Table 4:** Comparative analysis of physiological parameters: luteal versus follicular phase performance A Wilcoxon signed-rank test was used for comparison and was considered statistically significant at a p-value less than 0.05.

Luteal versus follicular phase comparison	Negative ranks	Positive ranks	Ties	Z-statistic	p-value
Cardiac efficiency	5	95	0	-8.625	0.000^*^
Total work done	3	97	0	-8.374	0.000^*^
Work done per min	46	43	11	-0.674	0.500^*^

Similarly, total work done demonstrated a pronounced variation, with 97 observations showing improved performance during the luteal phase and merely 3 observations indicating lower work output. The Z-statistic of -8.374 and a p-value of 0.000 further confirm a statistically significant difference in total work done between the two phases.

In contrast, work done per minute exhibited a more balanced distribution, with 46 negative ranks, 43 positive ranks, and 11 ties. The Z-statistic of -0.674 and a p-value of 0.500 indicate no statistically significant difference in work done per minute between the luteal and follicular phases. This suggests that while cardiac efficiency and total work done vary considerably across menstrual cycle phases, the rate of work performance remains consistent.

## Discussion

The current bicycle ergometric investigation provides a comprehensive exploration of physiological performance variations across distinct menstrual cycle phases, offering unprecedented insights into the intricate neuroendocrine mechanisms governing female physiological adaptation [3,4]. The multifaceted analysis revealed complex inter-dynamics between hormonal fluctuations and performance metrics, challenging existing paradigms of female physiological functioning.

Hormonal modulation of cardiac efficiency

The most remarkable finding emerged in cardiac efficiency, demonstrating a statistically significant 41.26% increase during the mid-luteal phase (p = 0.000). This substantial variation aligns with contemporary research investigating hormonal influence on cardiovascular dynamics [4,6]. The observed enhancement can be attributed to the differential neuroendocrine milieu characteristic of the luteal phase, where elevated progesterone and estrogen concentrations potentially modulate cardiovascular responsiveness and metabolic efficiency.

Neurophysiological performance mechanisms

The intricate interplay between hormonal variations and physiological performance manifested through nuanced alterations in total work done and cardiac efficiency. The remarkable observation of 97 participants exhibiting enhanced performance during the luteal phase suggests a profound hormonal influence on neuromuscular recruitment and metabolic adaptability [7,8,11]. These findings corroborate emerging research highlighting the complex endocrine-performance interface.

Metabolic and cardiovascular adaptations

Physiological plasticity was evident in the consistent work done per minute across menstrual cycle phases, indicating a robust neurological regulatory mechanism. This stability suggests an inherent physiological compensation strategy, whereby the body maintains consistent instantaneous work output despite significant hormonal fluctuations [3,11].

Comparative performance dynamics

The comparative analysis revealed statistically significant differences in cardiac efficiency and total work done, with minimal variations in work rate. This nuanced pattern suggests a sophisticated physiological adaptation mechanism that preserves core performance parameters while allowing flexibility in overall work capacity. The Wilcoxon signed-rank test’s results (Z-statistic of -8.625 for cardiac efficiency, p = 0.000) provide robust statistical validation of these observations.

Interdisciplinary implications

The findings transcend traditional exercise physiology boundaries, intersecting with reproductive endocrinology, occupational health, and performance optimization. The demonstrated physiological variations challenge historical perspectives that often marginalized menstrual cycle-related performance dynamics, offering a more comprehensive understanding of female physiological plasticity [1,2,9].

Neuroendocrine interaction hypothesis

Our results substantiate an emerging hypothesis of dynamic neuroendocrine interactions governing physiological performance. The observed performance variations likely result from complex interactions between hormonal fluctuations, metabolic adaptations, and neurophysiological regulatory mechanisms [4,5,7].

Clinical significance

The clinical implications of these findings extend beyond mere physiological observations, offering potentially transformative insights for occupational health, exercise prescription, and women’s performance optimization. Understanding menstrual cycle-related physiological variations can inform targeted intervention strategies across multiple domains.

In occupational settings, these findings suggest the potential for personalized performance management strategies that account for individual physiological variations. Healthcare providers and occupational health professionals could develop nuanced approaches that acknowledge cyclical performance fluctuations, potentially mitigating productivity challenges associated with menstrual cycle-related symptoms [13,14].

The observed cardiac efficiency variations have profound implications for exercise prescription and athletic training. Sports medicine practitioners and physiotherapists could design periodized training programs that strategically leverage the physiological advantages observed during different menstrual cycle phases [9,15]. Such approaches could optimize training intensity, recovery strategies, and performance outcomes for female athletes and physically active individuals.

Moreover, the research contributes to a more comprehensive understanding of women’s physiological adaptability, challenging historical paradigms that often overlooked menstrual cycle-related variations. By quantifying performance metrics across different cycle phases, the study provides empirical evidence supporting individualized performance management strategies.

The findings also hold significant relevance for reproductive health research. The demonstrated physiological variations suggest complex interactions between hormonal fluctuations and systemic performance, potentially offering insights into broader reproductive health considerations [4,5].

Strengths of the study

The study’s primary strength lies in its rigorous methodological approach and comprehensive experimental design. By utilizing a standardized bicycle ergometer and implementing precise measurement protocols, the research achieved high-resolution physiological assessment across multiple parameters. The large sample size of 100 participants enhances the study’s statistical power and generalizability. Careful participant selection, controlling for age and anthropometric characteristics, minimizing potential confounding variables. The use of multiple measurement points and the Wilcoxon signed rank test enabled robust statistical analysis, providing nuanced insights into menstrual cycle-related physiological variations. The research’s novel contribution stems from its holistic approach, simultaneously examining cardiac efficiency, total work done, and work rate. This multifaceted assessment provides a more comprehensive understanding of physiological performance variations compared to previous studies focusing on isolated parameters.

Limitations

The research was confined to a specific demographic of young women in Chennai, potentially limiting broader generalizability. The age range of 18-25 years might not represent the full spectrum of physiological responses across different life stages. The study’s cross-sectional design precludes direct observation of individual longitudinal variations. While statistically significant trends were identified, individual physiological responses can exhibit substantial variability. The bicycle ergometer, while providing precise measurements, represents a controlled laboratory environment. Real-world work performance involves complex, multifactorial interactions that might not be fully captured by this experimental setup. Additionally, the study did not comprehensively assess hormonal levels or account for individual variations in menstrual cycle regularity. Factors such as oral contraceptive use, underlying health conditions, and lifestyle variables were not extensively explored.

Recommendations

Future research should focus on comprehensive, multifaceted investigations that address current methodological limitations. Researchers are recommended to implement longitudinal study designs incorporating comprehensive endocrinological assessments, expand demographic diversity beyond current participant characteristics, and develop standardized protocols for menstrual cycle performance evaluations. Advanced research should integrate sophisticated hormonal biochemical analyses with sophisticated ergometric measurements, exploring potential causal mechanisms underlying performance variations. Interdisciplinary collaborative approaches combining exercise physiology, reproductive endocrinology, and occupational health perspectives will provide a more nuanced understanding of menstrual cycle-related physiological dynamics. Strategic research priorities should emphasize developing evidence-based, personalized performance optimization strategies that acknowledge individual physiological variations across different menstrual cycle phases.

## Conclusions

The present study provides compelling evidence of significant physiological performance variations across menstrual cycle phases, demonstrating notable increases in cardiac efficiency and total work done during the mid-luteal phase. These findings underscore the complex interplay between hormonal dynamics and physiological performance, challenging conventional perspectives and offering nuanced insights into women’s physiological adaptability. By quantifying performance metrics across different cycle phases, the research contributes critical empirical evidence supporting individualized performance management strategies. The statistically significant variations in cardiac efficiency and total work done highlight the importance of understanding cyclical physiological changes, with potential implications for occupational health, athletic training, and reproductive health research. While acknowledging the study’s limitations, the findings represent a significant step toward a comprehensive understanding of menstrual cycle-related performance dynamics, emphasizing the need for continued research in this critical domain.
